# *Culex pipiens,* an Experimental Efficient Vector of West Nile and Rift Valley Fever Viruses in the Maghreb Region

**DOI:** 10.1371/journal.pone.0036757

**Published:** 2012-05-31

**Authors:** Fadila Amraoui, Ghazi Krida, Ali Bouattour, Adel Rhim, Jabeur Daaboub, Zoubir Harrat, Said-Chawki Boubidi, Mhamed Tijane, Mhammed Sarih, Anna-Bella Failloux

**Affiliations:** 1 Institut Pasteur du Maroc, Laboratoire des Maladies Vectorielles, Casablanca, Maroc; 2 Institut Pasteur Tunis, Université Tunis-El Manar, Laboratoire d’Epidémiologie et de Microbiologie vétérinaire, Service d’Entomologie Médicale, Tunis-Belvédère, Tunisie; 3 Institut National Agronomique de Tunisie, Université Carthage, Tunis-Mahrajène, Tunisie; 4 Direction d’Hygiène du Milieu et de la Protection de l’Environnement, Ministère de la Santé Publique en Tunisie, Bab Saâdoun, Tunis, Tunisie; 5 Institut Pasteur d’Alger, Unité d’Entomologie Médicale, Service d’Eco-épidémiologie parasitaire et génétique des populations, Alger, Algérie; 6 Faculté des Sciences, Laboratoire de Biochimie et Immunologie, Rabat, Maroc; 7 Institut Pasteur, Department of Virology, Arboviruses and Insect Vectors, Paris, France; The University of Texas Medical Branch, United States of America

## Abstract

West Nile fever (WNF) and Rift Valley fever (RVF) are emerging diseases causing epidemics outside their natural range of distribution. West Nile virus (WNV) circulates widely and harmlessly in the old world among birds as amplifying hosts, and horses and humans as accidental dead-end hosts. Rift Valley fever virus (RVFV) re-emerges periodically in Africa causing massive outbreaks. In the Maghreb, eco-climatic and entomologic conditions are favourable for WNV and RVFV emergence. Both viruses are transmitted by mosquitoes belonging to the *Culex pipiens* complex. We evaluated the ability of different populations of *Cx. pipiens* from North Africa to transmit WNV and the avirulent RVFV Clone 13 strain. Mosquitoes collected in Algeria, Morocco, and Tunisia during the summer 2010 were experimentally infected with WNV and RVFV Clone 13 strain at titers of 10^7.8^ and 10^8.5^ plaque forming units/mL, respectively. Disseminated infection and transmission rates were estimated 14–21 days following the exposure to the infectious blood-meal. We show that 14 days after exposure to WNV, all mosquito st developed a high disseminated infection and were able to excrete infectious saliva. However, only 69.2% of mosquito strains developed a disseminated infection with RVFV Clone 13 strain, and among them, 77.8% were able to deliver virus through saliva. Thus, *Cx. pipiens* from the Maghreb are efficient experimental vectors to transmit WNV and to a lesser extent, RVFV Clone 13 strain. The epidemiologic importance of our findings should be considered in the light of other parameters related to mosquito ecology and biology.

## Introduction

West Nile virus (WNV) and Rift Valley fever virus (RVFV) are two arthropod-borne RNA viruses transmitted mainly by mosquitoes. WNV (Flaviviridae family, *Flavivirus* genus) was first isolated in Uganda in 1937 [1] and is now the most widely distributed arbovirus through the world [2]. This virus is maintained and amplified in nature within an enzootic transmission cycle, among birds and mosquitoes, whereas humans and mammals including horses are accidental dead-end hosts (reviewed in [3]). West Nile fever (WNF) was not of public health concern until its unexpected emergence outside its native range of distribution. In the early 1990s, outbreaks began to occur more frequently, especially in the Mediterranean Basin. In the Maghreb, human cases of meningo-encephalitis with fatalities occurred in Algeria in 1994 [4] and in Tunisia in 1997 [5] whereas epizootics in horses were reported in Morocco in 1996 [6]. In the following years, cases were reported again: in Tunisia in 2003 [7] and 2008 [8] and in Morocco in 2003 and 2010 [9], [10] indicating that WNV is still circulating in the region. RVFV (*Phlebovirus* genus, *Bunyaviridae* family), first identified in Kenya in 1931 [11] was responsible of numerous outbreaks affecting livestock and occasionally, humans in Sub-Saharan Africa. The first emergence of Rift Valley fever (RVF) outside Africa occurred in 2000–2001 in Saudi Arabia and Yemen [12]. Illegal trading of livestock between RVF-endemic regions with their bordering countries stresses the risk for RVF emergence in the Maghreb [13].

WNV and RVFV are transmitted by mosquitoes of the *Culex pipiens* complex including *Cx. pipiens* and *Cx. quinquefasciatus* which are ubiquitous mosquitoes in temperate and tropical regions, respectively. *Cx. pipiens* is the most widely distributed mosquito species in the Maghreb [14]–[17]. In this region, *Cx. pipiens* presents different eco-physiological characteristics. In urban areas, most *Cx. pipiens* populations colonize underground sites, are autogenous (lay first batch of eggs without taking a blood-meal), stenogamous (mate in confined spaces) and anthropophilic (biting preferentially humans) [18], [19]. Anautogenous populations (lay eggs after a blood meal) were also found in aboveground sites [20], [21]. Conversely, in rural areas, *Cx. pipiens* is anautogenous, stenogamous, anthropophilic or ornithophilic (biting preferentially birds) [22].

Determining the vectorial parameters influencing pathogen transmission is a critical step in understanding patterns of transmission and developing effective control interventions. The vector competence of *Cx. pipiens* is poorly defined in North Africa. In this paper, we show that populations of *Cx. pipiens* from the Maghreb are efficient experimental vectors of WNV and to a lesser extent, of RVFV.

**Table 1:** Characteristic of *Culex pipiens* sites sampled in Morocco, Algeria and Tunisia.

Country	City	Habitat	Breeding site	Autogenous (AU) or Anautogenous (AN)	Sample
Morocco	Casablanca	Urban	Underground	AU	M1_AU
				AN	M1_AN
	Mohammedia	Suburban	Underground	AU	M2_AU
				AN	M2_AN
Algeria	Timimoune	Urban	Underground	AU	A1_AU
				AN	A1_AN
	Chellal	Urban	Underground	AU	A2_AU
				AN	A2_AN
	Oued El Ksob	Suburban	Aboveground	AU	A3_AU
				AN	A3_AN
	Bechelga	Rural	Aboveground	AU	A4_AU
				AN	A4_AN
Tunisia	Tabarka	Urban	Aboveground	AU	T1_AU
				AN	T1_AN
	Nefza	Rural	Aboveground	AU	T2_AU
				AN	T2_AN

**Figure 1:**
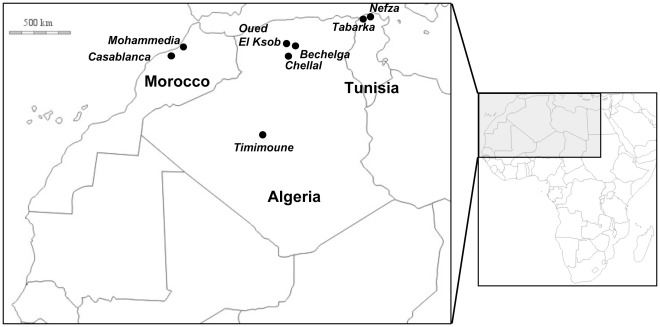
Localization of *Culex pipiens* samples collected in 2010 in the Maghreb (Morocco, Algeria and Tunisia).

## Materials and Methods

### Ethics Statement

No specific permissions are required for the field activities which do not involve endangered or protected species. The field sites are not privately-owned or protected properties. The Institut Pasteur in Morocco, Algeria and Tunisia are public institutions of health and scientific research placed under the supervision of the Ministry of Health. In this frame, they are involved in vector control activities which authorize them to operate without any specific permission for access to breeding sites and mosquito collections. According to European regulations, manipulations of pathogens belonging to the group 3 (WNV and RVFV) were carried out in biosafety level (BSL) 3 facilities.

**Figure 2:**
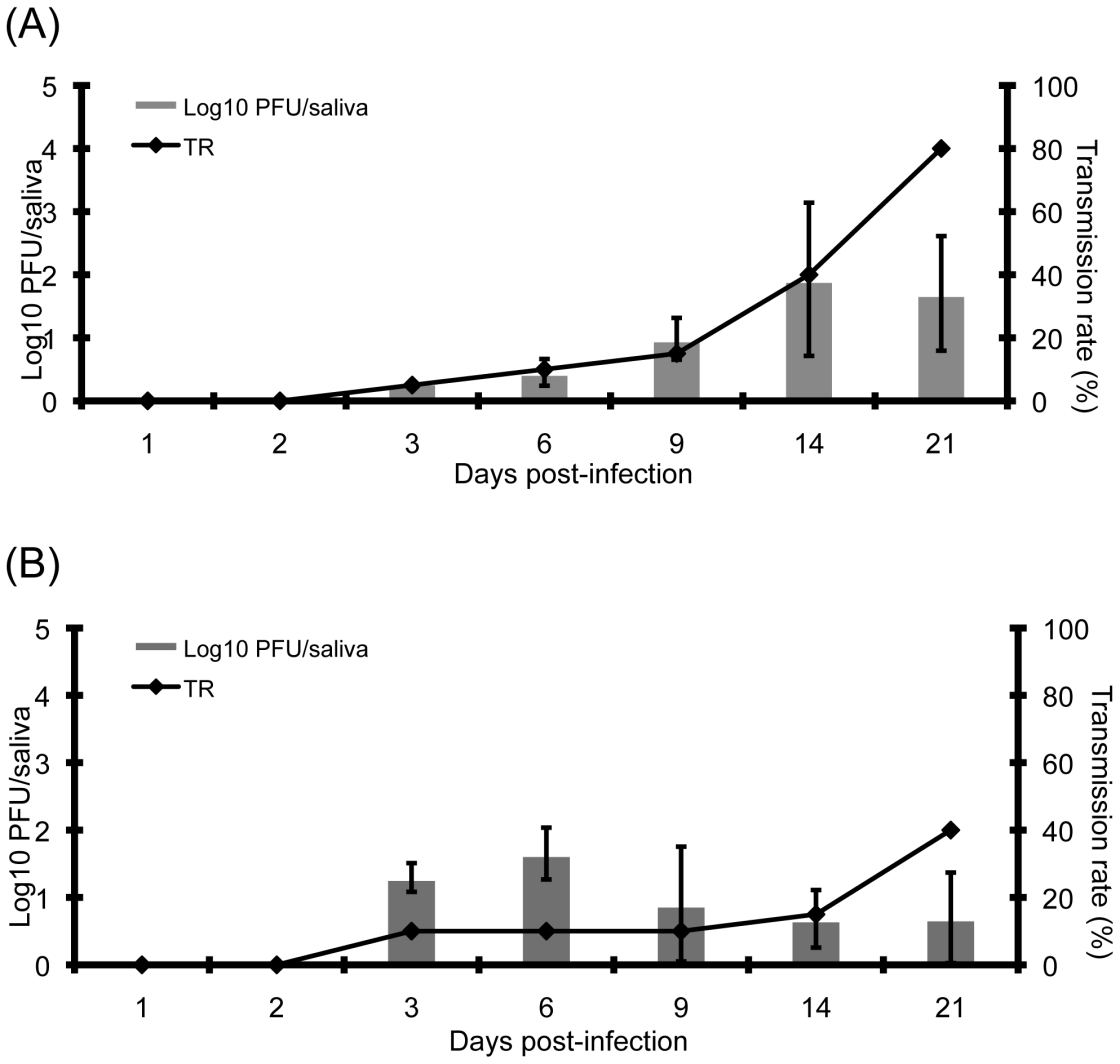
Transmission rate and mean titer of infectious viral particles present in saliva of *Culex pipiens* at different days after ingestion of an infectious blood-meal containing WNV (A) and RVFV (B). We exposed a *Culex pipiens* colony, Tabarka (Tunisia) to an infectious blood-meal containing 10^7.8^ PFU/mL of WNV or 10^8.5^ PFU/mL of RVFV. At day 3, 6, 9, 14 and 21 post-infection, 20 females were analyzed. Saliva were collected using the forced salivation technique. After removing wings and legs, the proboscis of mosquitoes was inserted into 20 µL tip filled with 5 µL of Fetal Bovine Serum (FBS). After 45 min, medium containing the saliva was collected into 45 µL of L15 medium. The number of infectious particles per saliva was estimated by titration on Vero cells and expressed as log_10_PFU/saliva. Lines refer to TR and bars to Log10 pfu/saliva.

### Mosquitoes

Eight populations of *Cx. pipiens* were sampled in different sites in Algeria (4), Morocco (2) and Tunisia (2) during summer 2010 (Table 1, Figure 1). Sites were classified according to the habitat (urban, suburban or rural) and the type of breeding site (aboveground and underground). The mosquitoes were collected as larvae and reared until imago stage. Batches of 200 larvae were reared in pans containing 1 liter of water supplemented with 1–2 of yeast tablets. This standardized rearing procedure allows obtaining females of similar size, making them likely to take equal quantities of blood and to ingest a similar number of viral particles. Placed on cages, adults were fed on 10% sucrose at 28±1°C with 80% relative humidity and a 16 h∶8 h photoperiod. Females able to lay eggs without any blood-meal were qualified as autogenous (AU) and those which required a blood-meal as anautogenous (AN). Thus from each of the 8 F0 collections, two F1 strains were obtained: AU and AN (Table 1). F1 adults were tested for their susceptibility to WNV and RVFV Clone 13 strain. The parameters of vector competence used for field-collected samples were defined using the F6 generation established from a sample collected in Tabarka in 2010 (Tunisia). This strain is adapted to laboratory conditions and feeds well on artificial blood-meals.

**Figure 3:**
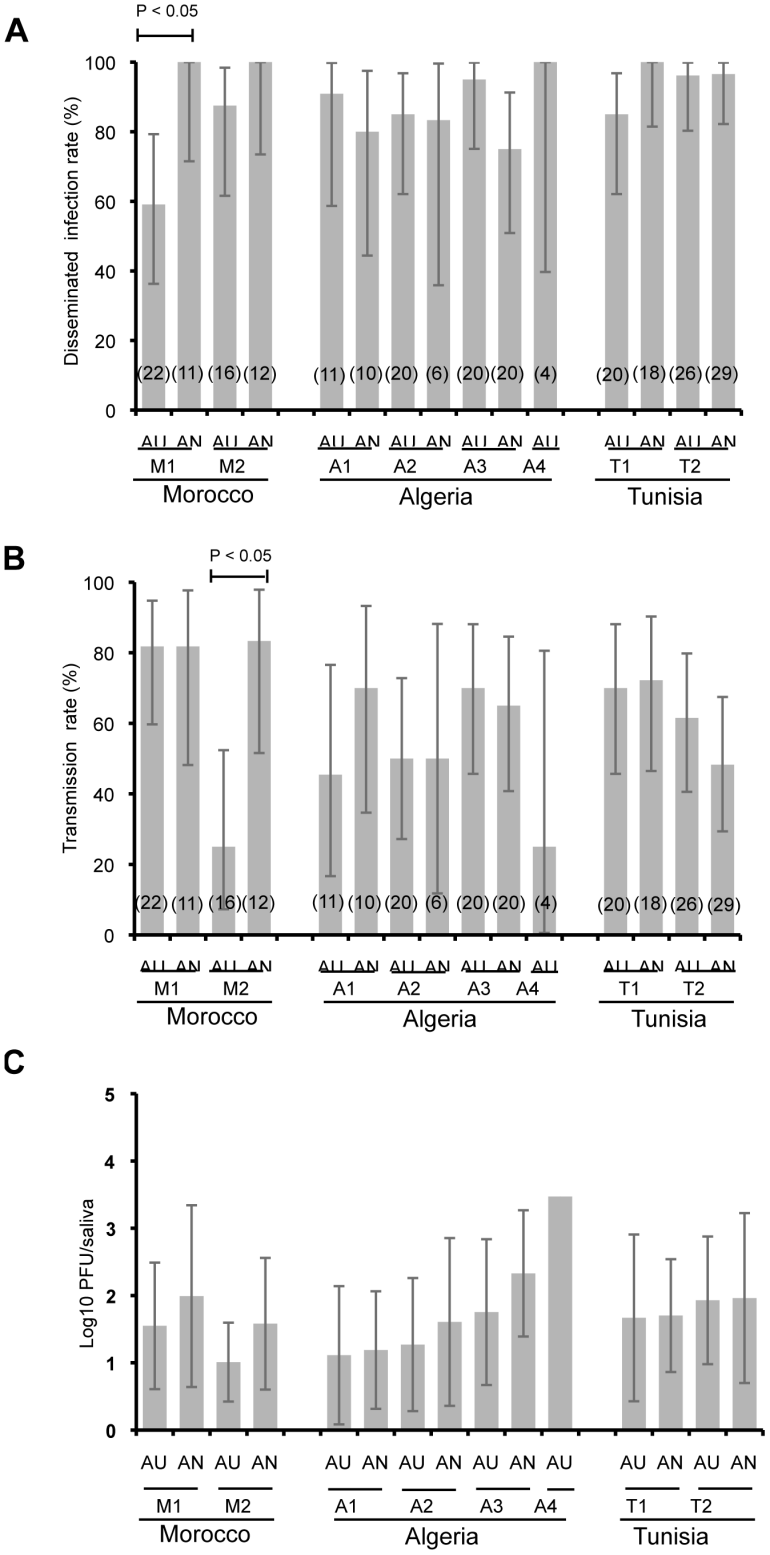
Disseminated infection rate (A), Transmission rate (B) and mean titer of infectious viral particles present in saliva (C) of *Culex pipiens* challenged with WNV. F1 mosquitoes (autogenous AU and anautogenous AN) were orally challenged with WNV at a titer of 10^7.8^ PFU/mL using an artificial feeding system. After completion of the blood-meal, mosquitoes were maintained in BSL-3 insectaries at 28°C. At day 14 pi, saliva was collected from surviving females using the forced salivation technique. The number of infectious viral particles present in saliva was estimated by plaque assay on Vero cells. After salivation, females were tested for the presence of WNV on head squashes by IFA. p<0.05, Fisher’s exact test. In brackets, the number of mosquitoes tested. Error bars show the confidence interval (95%) for DIR and TR, and the standard deviation for Log10 pfu/saliva.

Except cases mentioned above, no significant difference in DIR, TR and number of infectious particles in saliva was found 14 days after exposure to a WNV-infectious blood-meal whatever mosquitoes are autogenous or anautogenous.

### Viruses

The WNV strain was isolated from a horse in Camargue (France) in 2000 [23]. After 4 passages on Vero cells, the WNV stock was produced on *Ae. albopictus* cells C6/36 [24]. The RVFV is the avirulent strain Clone 13 isolated from a human case in Bangui (Central African Republic) in 1974 [25]. After 8 passages on Vero cells, the RVFV stock was produced on C6/36 cells. Viral stocks were stored at −80°C in aliquots until use.

**Figure 4:**
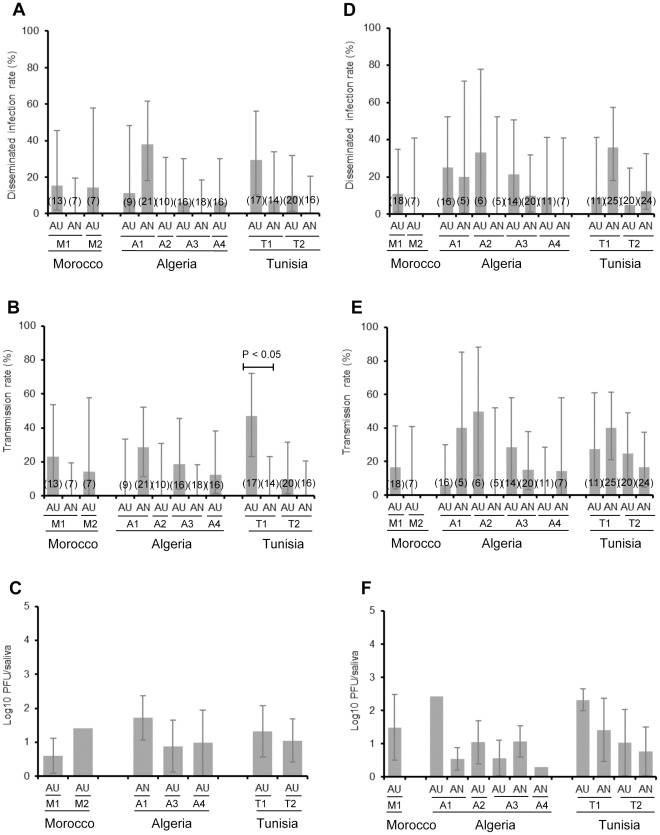
Disseminated infection rate, Transmission rate and mean titer of infectious viral particles present in saliva of *Culex pipiens* at day 14 (A,B and C) and 21 (D,E and F) post-infection with RVFV. F1 mosquitoes (autogenous AU and anautogenous AN) were orally challenged with RVFV at a titer of 10^8.5^ PFU/mL using an artificial feeding system. After completion of the blood-meal, mosquitoes were maintained in BSL-3 insectaries at 28°C. At day 14 pi and day 21 pi, saliva was collected from surviving females using the forced salivation technique. The number of infectious viral particles present in saliva was estimated by plaque assay on Vero cells. After salivation, females were tested for the presence of RVFV on head squashes by IFA. In brackets, the number of mosquitoes tested. Error bars show the confidence interval (95%) for DIR and TR, and the standard deviation for Log10 pfu/saliva.

### Oral Infections of Mosquitoes

Infection assays were performed with 7 day-old F1 females which were allowed to feed for 30 min through a pig intestine membrane covering the base of a glass feeder containing the blood-virus mixture maintained at 37°C. The infectious meal was composed of a viral suspension (1∶3) diluted in washed rabbit erythrocytes isolated from arterial blood collected 24 h before the infection [26]. The ATP was added as a phagostimulant at a final concentration of 5×10^−3^ M. Virus titer in the blood-meal was at 10^7.8^ PFU/mL for WNV and 10^8.5^ PFU/mL for RVFV. Fully engorged females were transferred in cardboard containers and maintained with 10% sucrose at 28±1°C for 14–21 days.

### Saliva Collection

After the incubation period, saliva was collected using the forced salivation technique. Briefly, mosquitoes were chilled, their wings and legs removed and the proboscis was inserted into 20 µL tip filled with 5 µL of Fetal Bovine Serum (FBS). After 45 min, medium containing the saliva was expelled into 1.5 mL tube containing 45 µL of Leibovitz L15 medium. For the colony from Tabarka, saliva was collected at different days: 1, 2, 3, 6, 9, 14 and 21 days after the exposure to the infectious blood-meal.

### Virus Titration

The number of infectious particles per saliva was estimated by titration on Vero cells and expressed as log_10_PFU/saliva. Briefly, six-well plates containing confluent monolayers of Vero cells were infected with serial 10-fold dilutions of virus. Cells were incubated for four days (WNV) or five days (RVFV) under an overlay consisting of Dulbecco’s MEM (DMEM), 2% Fetal Bovine Serum, 1% antibiotic-antimycotic mix (Invitrogen, Gibco) and 1% agarose at 37°C. The lytic plaques were counted after staining with a solution of crystal violet (0.2% in 10% formaldehyde and 20% ethanol). The transmission rate (TR) corresponds to the proportion of mosquitoes whose saliva contains infectious viral particles among mosquitoes presenting a disseminated infection.

### Female Status Analyzed by Immunofluorescent Assay

After salivation, females were sacrified and tested for the presence of WNV and RVFV viruses on their head squashes by immunofluorescence assay (IFA) [27]. The presence of virus in head squashes results from the viral dissemination in the hemocele after passing through the midgut. The disseminated infection rate (DIR) corresponds to the proportion of mosquitoes with infected head squashes among tested mosquitoes.

### Statistical Analysis

The Fisher’s exact test was used for comparisons of rates (DIR and TR) and the Kruskall-Wallis test for comparisons of mean titers of infectious viral particles in saliva using the STATA software (StataCorp LP, Texas, USA).

## Results

### Susceptibility to WNV

The colony *Cx. pipiens* from Tabarka (Tunisia) was firstly tested to determine the day post-infection (pi) to collect mosquito saliva and assess TR of field-collected samples (Figure 2A). WNV started to be detected in the saliva at day 3 pi with a TR of 5% which increased slightly until day 9 pi. At day 14 pi, 40% of saliva tested were infected and the number of infectious particles in saliva reached its maximum (mean ± standard deviation: 1.9±1.2 log_10_PFU). At day 21 pi, TR continued to increase until 80% and the number of infectious particles started to slightly decrease to 1.7±0.9 log_10_PFU. Thus, day 14 pi was considered to estimate DIR and TR when mosquitoes were challenged with WNV.

Fourteen days after exposure to WNV, all mosquito strains tested developed a disseminated infection and were able to deliver virus through saliva (Figure 3). Strains presented DIRs ranging from 59.1% to 100% (Figure 3A) and TRs varying from 25% to 83.3% (Figure 3B). When comparing autogenous (AU) and anautogenous (AN) mosquitoes from a same collection site, DIRs and TRs were comparable (Fisher’s exact test: p>0.05) except for two strains from Morocco: strain M1 for DIR (Fisher’s exact test: p = 0.02) and strain M2 for TR (Fisher’s exact test: p = 0.01). The number of infectious particles in saliva varied from 1.0±0.6 log_10_PFU to 3.5 log_10_PFU (Figure 3C). When comparing the number of infectious particles in saliva between AU and AN mosquitoes from a same collection site, no significant difference was found (Kruskall-Wallis test: p>0.05).

### Susceptibility to RVFV

With the colony *Cx. pipiens* from Tabarka, RVFV started to be detected at day 3 pi with a TR of 10% and 1.3±0.2 log_10_PFU in saliva (Figure 2B). TR remained steady until day 14 pi and reached a maximum of 40% at day 21 pi. The number of infectious particles was at its highest level at day 6 pi with 1.6±0.4 log_10_PFU and decreased from day 9 to day 21 pi. As a compromise, we chose to estimate DIR and TR at day 14 and day 21 pi when mosquitoes were exposed to RVFV.

Fourteen days after exposure to RVFV, 69.2% (9 strains among 13 tested) of mosquito strains developed a disseminated infection with DIRs ranging from 6.2% to 38.1% (Figure 4A). Among strains exhibiting positive DIRs, 77.8% (7/9) of strains had virus detected in saliva with TRs varying from 10% to 47.1% (Figure 4B). Thus, two strains, A1_AU and T1_AN were not capable to get infected saliva after the dissemination of the virus from the midgut. When available, comparisons between AU and AN mosquitoes from a same collection site, did not show any significant difference of DIRs and TRs (Fisher’s exact test: p>0.05) except for the T1 strain from Tunisia for TR (Fisher’s exact test: p = 0.004). The number of infectious particles in saliva varied from 0.6±0.5 log_10_PFU to 1.7±0.7 log_10_PFU (Figure 4C). Most infectious saliva came from AU mosquitoes.

At day 21 pi, 78.6% (11 strains among 14 tested) of mosquito strains developed a disseminated infection with DIRs varying from 5% to 36% (Figure 4D). 91% mosquito strains were able to excrete infectious saliva with TR ranging from 6.2% to 50% (Figure 4E). When available, comparisons between AU and AN mosquitoes from a same collection site, did not show any significant difference of DIRs and TRs (Fisher’s exact test: p>0.05). 85.7% of strains were capable to deliver infectious particles in saliva with a number varying from 0.3 log_10_PFU to 2.4 log_10_PFU (Figure 4F). Thus, increasing the extrinsic incubation period from 14 days to 21 days increased the proportion of mosquito strains with positive DIRs and TRs. Moreover, the number of infectious viral particles in saliva increased concomitantly even if not statistically validated (Wilcoxon rank-sum test: p>0.05). Autogenous mosquitoes were more capable to ensure the viral dissemination and transmission. At day 14 pi, 61.5% of samples capable to ensure viral dissemination and transmission were AU mosquitoes and 38.5% were AN mosquitoes. At day 21 pi, 57.1% of samples able to ensure dissemination and transmission were AU mosquitoes and 42.9% were AN mosquitoes.

When considering each mosquito strain and comparing the DIRs estimated 14 days after infection with WNV and RVFV, significant differences were found with highest DIRs obtained with WNV (Fisher’s exact test: P<0.05). When analyzing the TRs estimated 14 days after infection with WNV and RVFV, significant differences were obtained for 4 strains among 13 (Fisher’s exact test: P<0.05). However, the number of infectious particles in saliva was not significantly different when examining each mosquito strain infected with WNV and RVFV (Wilcoxon rank-sum test: p>0.05).

## Discussion

*Culex pipiens* is the most widely distributed mosquito species in the Maghreb and is suspected to be involved in WNV and RVFV transmission. Using experimental infections, we showed that *Cx. pipiens* populations collected in Algeria, Morocco and Tunisia were highly susceptible to infection and readily to transmit WNV and to a lesser extent, RVFV.

To be transmitted to a vertebrate host, an arbovirus must be able to reach and infect the salivary glands. After feeding on a viremic vertebrate host, the ingested virus must penetrate into the midgut epithelial cells, replicates and subsequently, escape from the midgut. The virus disseminates within the body cavity infecting tissues and organs including salivary glands. Infectious viral particles are injected into a new vertebrate host along with saliva. Barriers to the overall sequence leading to transmission are described: the midgut and the salivary glands (reviewed in [28]). The efficiency of these barriers determines the level of mosquito vector competence. For both viruses tested, WNV and RVFV, the time interval between the ingestion of a viremic blood-meal and the ability of a mosquito to transmit a pathogen, described as the extrinsic incubation period (EIP) was 3 days with *Cx. pipiens* from Tabarka (Tunisia).

When exposed to an infectious blood-meal containing WNV, all mosquito strains collected in 8 different sites in the Maghreb, were capable to ensure efficient viral dissemination and transmission at day 14 pi. Our findings are in line with the predominant role of *Cx. pipiens* in the transmission of WNV. DIRs varied from 59% to 100%, and TRs from 25% to 100%. The number of viral particles delivered with saliva was up to ∼ 12800 particles. Vector competence is mainly influenced by viral dose, incubation period and temperature. We used a viral titre of 10^7.8^ PFU/mL and an incubation temperature of 28°C, both factors affecting viral dissemination [29]. Indeed, the minimal infectious doses required to infect *Cx. pipiens* should be greater than 10^5.0^ PFU/mL [30] and high temperatures increase viral replication [31]. Previous studies have shown spatial variations in WNV vector competence of *Cx. pipiens* [32]–[34]. We also observed geographic variations in vector competence without assignment of high performances to a given country or a collection site.

We used for RVFV, the Clone 13 which is a naturally attenuated strain with a deletion of 70% of the gene NSs playing a key role in the pathogenesis of RVFV [35], [36]. It has been shown that this deletion could affect viral replication in mosquitoes. It has been shown that dissemination was higher when exposed mosquitoes to a virulent RVFV [37]. We found that 14 days after exposure to RVFV, 69.2% of mosquito strains were able to develop a disseminated infection with DIRs up to 38.1%, values higher than those previously found for *Cx. pipiens* populations from Tunisia [38] but lower than DIRs for laboratory colonies of *Cx. pipiens* [39]. Most strains (77.8%) were able to transmit the virus with up to ∼ 620 viral particles detected in saliva. The midgut infection was the most important barrier to viral dissemination [40]. The moderate ability of *Cx. pipiens* to transmit RVFV is mostly due to the inefficiency of virions to escape from midgut epithelial cells to infect secondary target organs [41]. When increasing the incubation period up to 21 days, 78.6% of mosquito strains develop a disseminated infection and 91% were able to deliver infectious particles in saliva. Thus, *Cx. pipiens* with disseminated infection that did not have infectious saliva at day 14 pi may have viral infections to develop a week later [42]. Moreover, the midgut barrier appears to be operating by delaying the release of the virus into the general cavity of *Cx. pipiens* infected with RVFV [41]. A sporadic dissemination of virus from the midgut was likely to operate rather than a complete blockade of the virus inside the midgut epithelial cells.

Our strains contain a mix of autogenous (AU) and anautogenous (AN) mosquitoes. The two forms are thought to have different vector competences (reviewed in [43]). Indeed, we found evidence that when challenged with RVFV, AU mosquitoes were predominantly capable to ensure the viral dissemination and transmission, 14 days after the exposure to the infectious blood-meal (see Figure 4C). Surprisingly, AN mosquitoes were characterized by a delay in RVFV transmission; AN populations were more likely to transmit 21 days after feeding on an infectious blood-meal (see Figure 4F). We suggested that epizootic outbreaks of RVF can be initiated by *Aedes* or *Ochlerotatus* mosquitoes which are present in high densities in rural areas [14], [44], [45]. *Aedes* mosquitoes such as *Ae. vexans* in West Africa [46]–[49] capable to transmit the virus vertically to their offspring are likely to initiate the virus circulation. Subsequent epizootic outbreaks are associated with *Culex* mosquitoes. Based on their low vector competence, we hypothesized that AN mosquitoes in rural areas weakly take part to RVFV transmission. AU mosquitoes are more likely to serve as a bridge vector between animals and humans. A RVF cycle could then be initiated when AU mosquitoes reach densities high enough to trigger an epidemic/epizootic outbreak. The Maghreb region shares borders with RVF-endemic countries. In 2010, a severe outbreak has been reported in an extremely arid region of Mauritania close to borders with Morocco and Algeria [50]. Introduction of infected livestock raised concern for future emergences of RVF. Indeed, the RVF outbreaks in Egypt in 1977 and in Saudi Arabia in 2000 were caused by the trade of viremic animals [51], [52].

Like WNF, RVF could become epizootic and epidemic in the Maghreb if introduced. Unless vaccines are available and used on a very large scale to limit their expansion, both WNF and RVF will continue to be a critical issue for human and animal health. In a near future, protection of the public health will continue to rely on mosquito control. Further studies are required to understand the bio-ecology of *Cx. pipiens* and other mosquito vectors in the Maghreb.
